# Supplementary material for: Hybrid Raman and Laser-Induced Breakdown Spectroscopy for Food Authentication Applications
Source: Molecules. 2023 Aug 16;28(16):6087. doi: 10.3390/molecules28166087 (PMC10458226; doi:10.3390/molecules28166087)
Supplement: Supplementary file 1 [file molecules-28-06087-s001.zip › molecules-2519815-supplementary.pdf]

Hybrid Raman and laser-induced breakdown spectroscopy for food authentication applications

Sungho Shin[1,*], Iyll-Joon Doh[1], Kennedy Okeyo[2], Euiwon Bae[3], J. Paul Robinson[1,2], and Bartek Rajwa[4,*]

[1] *Department of Basic Medical Sciences, Purdue University, West Lafayette, IN 47907, USA*

[2] *Weldon School of Biomedical Engineering, Purdue University, West Lafayette, IN 47907, USA*

[3] *School of Mechanical Engineering, Purdue University, West Lafayette, IN 47907, USA*

[4] *Bindley Bioscience Center, Discovery Park, Purdue University, West Lafayette, IN 47907, USA*

[*] *Corresponding author: shin331@purdue.edu; brajwa@purdue.edu*

Supplementary Material

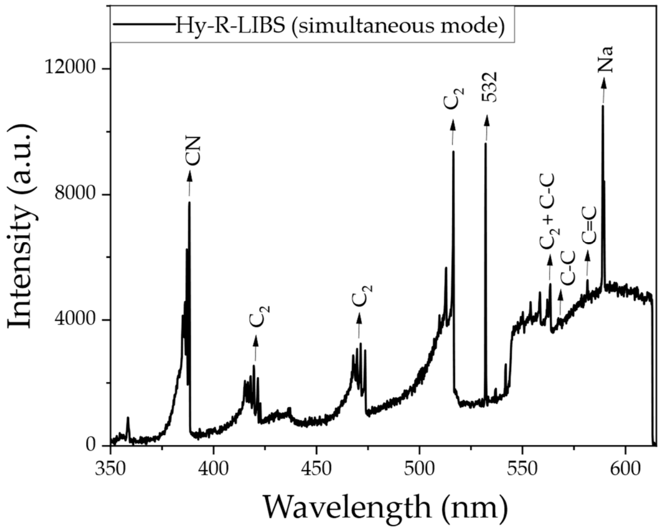

Figure S1. The average of ten simultaneous signals from Hy-R-LIBS system obtained from PS beads.

(a) **(b)**

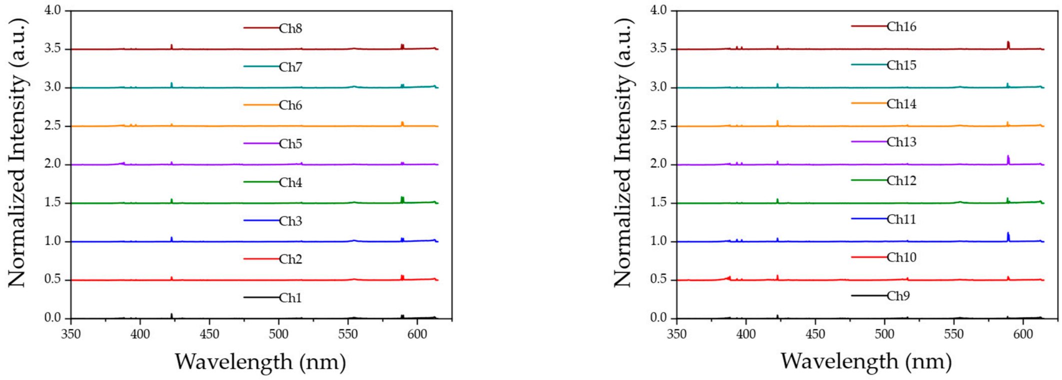

Figure S2. Averaged and normalized spectra of 16 cheese samples using the LIBS system for (a) 8 cheese samples, and (b) 8 other cheese samples.

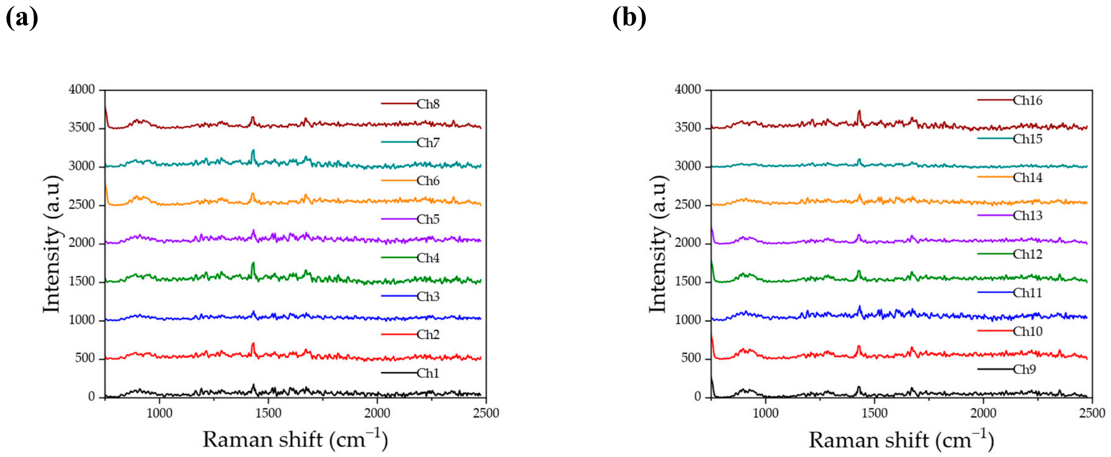

Figure S3. Averaged spectra of 16 cheese samples using the Raman spectroscopy system for (a) 8 cheese samples, and (b) other 8 cheese samples.

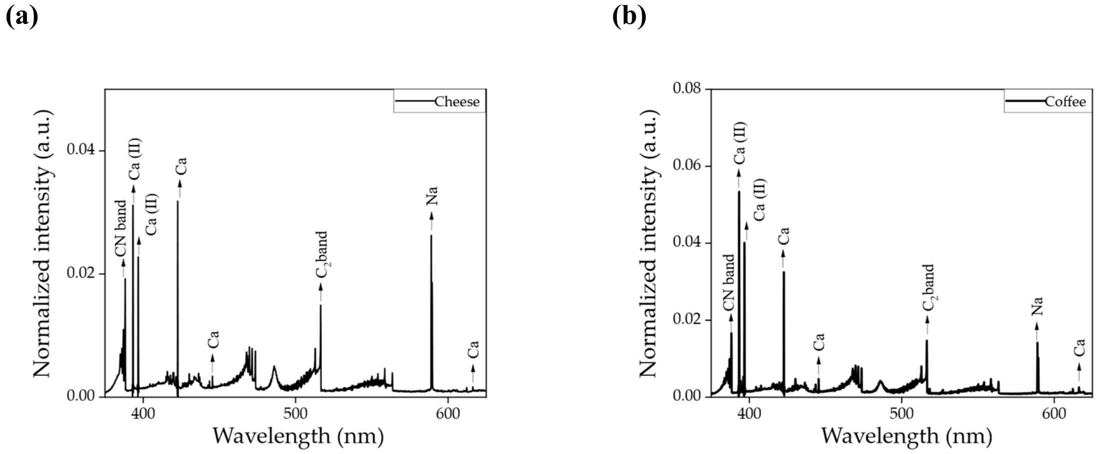

Figure S4. Averaged and normalized LIBS spectra of (a) all 16 cheese samples, and (b) all 7 coffee varieties obtained using a commercially available instrument (Z-900). Note that corresponding spectral range (350-625 nm) was selected.

(a) **(b)**

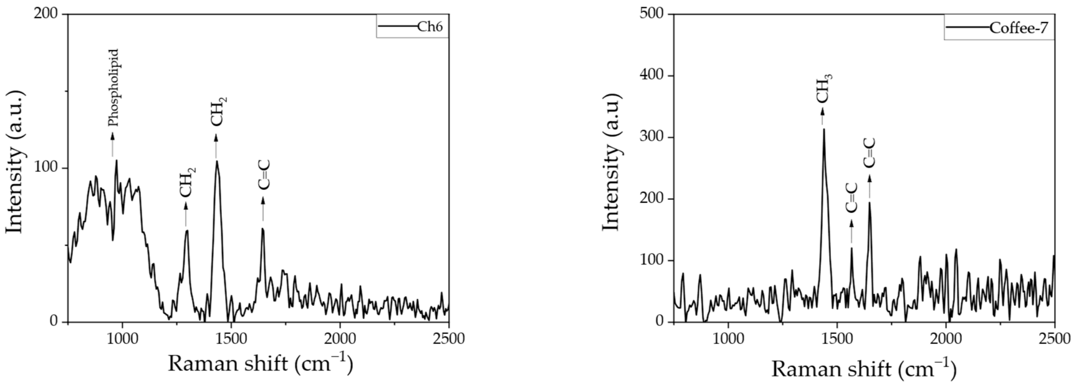

Figure S5. The averaged Raman spectrum for (a) a cheese sample (Ch6), and (b) a coffee sample (C7) collected using a commercially available instrument (Alpha300). Note that corresponding spectral range (750-2500 cm^{-1}) was selected.

		**Target class**															
		**Ch1**	**Ch2**	**Ch3**	**Ch4**	**Ch5**	**Ch6**	**Ch7**	**Ch8**	**Ch9**	**Ch10**	**Ch11**	**Ch12**	**Ch13**	**Ch14**	**Ch15**	**Ch16**
**Output class**	**Ch1**	**98**	0	0	1	0	0	0	0	1	0	0	0	0	0	0	0
	**Ch2**	0	**90**	0	1	0	0	2	1	1	2	3	0	0	0	0	0
	**Ch3**	0	0	**95**	1	0	0	2	0	0	0	1	0	0	0	1	0
	**Ch4**	2	3	1	**88**	0	0	0	1	2	2	1	0	0	0	0	0
	**Ch5**	0	0	2	0	**94**	1	0	0	1	1	0	0	0	0	0	1
	**Ch6**	0	0	0	0	1	**95**	0	0	0	0	0	0	1	2	1	0
	**Ch7**	0	0	2	3	3	2	**84**	0	1	0	0	0	1	3	1	0
	**Ch8**	0	1	0	2	0	1	3	**90**	1	0	0	0	2	0	0	0
	**Ch9**	0	0	0	1	0	0	2	2	**88**	3	0	0	0	1	2	1
	**Ch10**	0	1	0	1	2	1	1	0	1	**86**	1	0	1	2	1	2
	**Ch11**	0	1	0	0	0	0	2	1	0	2	**94**	0	0	0	0	0
	**Ch12**	0	0	0	0	0	0	0	0	0	0	0	**100**	0	0	0	0
	**Ch13**	0	2	0	0	0	0	0	3	0	0	0	0	**94**	0	0	1
	**Ch14**	0	1	0	2	0	0	2	1	2	0	0	0	0	**92**	0	0
	**Ch15**	0	0	0	0	0	0	2	1	0	3	0	0	0	0	**94**	0
	**Ch16**	0	1	0	0	0	0	0	0	2	1	0	0	1	0	0	**95**

Figure S6. An example of a confusion matrix calculated for all the tested cheese varieties using the ENET classifier. Note that this training/testing process was repeated 10 times to produce the results reported in Table 1.

		Target class						
		C1	C2	C3	C4	C5	C6	C7
Output class	C1	**93**	1	0	0	3	3	0
	C2	4	**91**	2	0	3	0	0
	C3	0	3	**95**	0	2	0	0
	C4	0	0	0	**100**	0	0	0
	C5	2	3	3	0	**89**	2	1
	C6	1	2	0	0	2	**95**	0
	C7	0	0	0	0	1	0	**99**

Figure S7. An example of a confusion matrix calculated for the tested coffee varieties using the ENET classifier. Note that this training/testing process was repeated 10 times to produce the results reported in Table 2.
